# Areca Nut Chewing: Initiation, Addiction, and Harmful Effects Emphasizing the Barriers and Importance of Cessation

**DOI:** 10.1155/2021/9967097

**Published:** 2021-06-03

**Authors:** I. A. Athukorala, W. M. Tilakaratne, R. D. Jayasinghe

**Affiliations:** ^1^Centre for Research in Oral Cancer, Faculty of Dental Sciences, University of Peradeniya, Peradeniya, Sri Lanka; ^2^Department of Oral and Maxillofacial Clinical Sciences, Faculty of Dentistry, University of Malaya, Kuala Lumpur, Malaysia; ^3^Department of Oral Medicine and Periodontology, Faculty of Dental Sciences, University of Peradeniya, Peradeniya, Sri Lanka

## Abstract

Areca nut is a highly addictive substance with carcinogenic properties and causes many harmful effects to the human body. Alkaloids are the major chemicals found in areca nut, and their mechanism of action has been studied for several years. About 600 million people around the world use areca nut, and its usage is higher in Asian countries. Areca nut chewers get used to the habit mainly due to social influence, stress, or lack of awareness. Some studies have reported a dependence syndrome related to areca nut chewing. Even though there are many ongoing research studies regarding tobacco addiction, less concern has been given to the areca nut addiction. There are only few studies related to areca nut cessation, and even those few cessation programs are mainly designed using tobacco cessation methodology. Sociocultural influence, behavioral factors, and accessibility are the main barriers to cessation. Behavior changing interventions proved to be more effective in areca nut cessation, but recently studies on pharmacological therapies are also emerging.

## 1. Introduction

Areca nut, also known as betel nut, is the seed of the palmaceous *Areca catechu* tree. It is a primary ingredient in betel quid. A definition was given for the word “quid” at a workshop held recently in Kuala Lumpur as “a substance, or mixture of substances, placed in the mouth or chewed and remaining in contact with the mucosa, usually containing one or both of the two basic ingredients, tobacco and/or areca nut, in raw or any manufactured or processed form” [1].

Approximately, 600 million people globally chew betel quid [2]. Most of them are from the Asia-Pacific region including Sri Lanka, India, Bangladesh, Myanmar, and Taiwan. Most of the betel chewers in other countries are also emigrants from these countries [3]. People use areca nut alone or in a betel quid comprising ingredients such as betel leaf, slaked lime, and tobacco [4]. Ingredients of betel quid can differ according to country, ethnicity, and personal choice [2]. The betel quid in Taiwan does not contain tobacco [5].

Areca nut is consumed in raw form or in processed forms (boiled, roasted, fermented, and sweetened) with or without tobacco in India and other South Asian countries. Areca nut pieces are also used as mawa with tobacco and slaked lime. Apart from being an ingredient in betel quid and mawa, areca nut is used in commercial products such as gutka and pan masala. Gutka and pan masala have all the ingredients of betel quid except for fresh betel leaves [6].

This is a comprehensive review on the areca nut addiction, its harmful effects, and various modalities that are being used to achieve cessation of the habit. This paper highlights the strengths and limitations of various areca nut cessation programs and present recommendations for the success of future cessation programs.

## 2. Causes for Commencement of Areca Nut Use

Factors associated with commencement of areca nut use have been investigated by several researchers [7–12]. Education level, ethnicity, cigarette smoking, alcohol use, signs of masochism, and social norms are the prominent factors discovered as causes for initiation of areca nut use [13]. Research conducted by Ghani et al. on factors which affect commencement and cessation of betel quid chewing behavior in Malaysian adults discovered gender, age, ethnicity, and history of smoking as the factors that influence the development and cessation of this habit [13]. According to a cross-sectional study done in Taiwan, most of the people who used to smoke and chew betel quid were less educated, middle aged males leading unhealthy lifestyles [14].

People older than 40 years were more likely to start chewing areca nut. Research that has been done by Yap et al. on Taiwanese adults also gave similar results [12]. However, a study done by Lee et al. on male inmate population in Taiwan calculated mean age at commencement of betel quid (BQ) chewing as 18.21 years [15]. Research done among the Gujarat community in the UK found that the mean age of first trying to use areca nut is 13 years [16]. This is supported by a study done by Murphy et al. among Chamorro and non-Chamorro Micronesian areca nut chewers which reported that the participants began chewing at a mean age of 17.77 years [17]. Accordingly, there is a controversy between different studies regarding the mean age of initiation of the habit.

According to Ghani et al., the habit of betel chewing was more prevalent among females in Malaysia [13], and this result is similar to findings of a study done in Cambodia [18]. However, studies done in India [19, 20], Taiwan [21], and Solomon Islands [10] indicated more prevalence of the aforementioned habit among males.

A study among the Taiwanese population has found that smokers are 10 times more likely to become areca nut chewers [21]. Lee et al. reported that the use of cigarette, alcohol, and BQ in male inmate population has shown a strong individual aggregation. Majority of the inmates claimed that they use all three substances as a habit [15]. Similar results have been observed in previous studies [9, 12, 22–24].

A study among adolescents in Taiwan cited that curiosity, keeping warm, and peer pressure are the reasons for starting the habit [25]. Another study done in India stated that “people initiate the habit as a way to pass the time, tension in the family and boredom” [26]. Sociocultural influence is also an important factor for initiation of the habit. During a pilot study done in Chitrakoot, India, majority of the users revealed that they got used to the habit due to pressure from the peers. Most of the females had developed the habit during pregnancy to improve the taste in their mouth. All the gutka users also revealed that one or more of their family members chewed gutka [27]. This fact is supported by Murphy et al. which reported that 50.9% of the chewers began chewing due to the influence of a family member. Moreover, 45.9% indicated that they started the habit because areca nut was readily available at their homes [17]. Recent study done by Sotto et al. on chewers in Guam also revealed that some of them were encouraged by their social group to chew [28].

## 3. Addictive Properties

Areca nut is considered as the fourth most commonly consumed psychoactive substance in the world preceded only by alcohol, nicotine, and caffeine containing beverages [29, 30]. Areca nut users report increased sense of well-being and stamina, euphoria, a soothing effect in the digestion, and protection of the mouth and gums [31]. In addition, areca nut chewing claimed to produce warm sensations of the body, sweating, salivation, palpitation and heightened alertness, and tolerance to hunger. All these neurological effects suggest that chemicals in areca nut affect autonomic nervous system, at various levels [32].

A significant increase in brain dopamine levels was observed in mice after arecoline was injected [33]. A monoamine oxidase-A (MAO-A) inhibition associated antidepressant effect could be observed within rat brains when dichloromethane fraction of areca nut was injected [34]. Winstock et al. have reported a dependence syndrome associated with areca nut chewing [16]. A study of six Asian countries, in specific groups such as Hunan men (a province of China), Malaysian women, and the Indonesian and Nepalese populations revealed that the incidence of betel quid dependence even exceeds that of alcohol dependence [35].

## 4. Carcinogenic Properties

The International Agency for Research on Cancer (IARC) has identified betel quid itself, with or without tobacco, as a group I carcinogen [4]. Areca nut used in any form causes oral cancer in humans [36]. Cancers of the esophagus, liver, pancreas, larynx, and lungs are also common among areca nut chewers [37]. Having slaked lime during betel chewing increases the pH of the oral cavity significantly, causing inflammation, and promotes oxidation of polyphenols in betel quid. Hence, reactive oxygen compounds which can promote carcinogenesis will be produced [38]. Alkaloids of areca nut are considered to be its main carcinogenic constituent [39]. Arecoline, arecaidine, guvacine, and guvacoline are 4 major alkaloids discovered during studies [40]. Apart from alkaloids, tannins and some of the polyphenols such as safrole, hydroxychavicol, and catechins are also proven as carcinogens [39]. Several research works conducted using animal models found that areca nut products and its derivatives can induce neoplastic changes in animals [31].

A majority of betel chewers show oral potentially malignant disorders (OPMDs) such as oral leukoplakia (OL), erythroplakia, or oral submucous fibrosis (OSF) [31, 41]. These conditions are considered as early and important indicators of oral cancer risk to an individual [42]. White patch or plaque on the oral mucosa which cannot be categorized as a definable disease is known as oral leukoplakia. It can be divided into several subtypes: homogenous, speckled, nodular, or verrucous leukoplakia. Red and white speckled lesions, also known as erythroplakia, have an increased risk for malignant transformation when compared to homogenous white lesions. Studies have shown that the quitting of areca nut use resolved 62% of leukoplakia, proposing that areca nut alone is an important etiological factor in the development of leukoplakia [41].

According to Johnson et al., “OSF is a chronic disorder characterized by fibrosis of the lining mucosa of the upper digestive tract involving the oral cavity, oro and hypo pharynx and the upper third of the esophagus” [43]. Chewing areca nut is considered as the single most important etiological factor for developing OSF [44–46]. A clear dose dependent relationship has been identified between both frequency and duration of chewing betel without tobacco and the development of OSF [47]. Exact mechanisms of development of OSF due to areca nut use are not fully understood. However, several studies have shown that the imbalance between collagen formation and breakdown due to increased tissue inhibitors of matrix metalloproteinases (TIMPs) and reduced matrix metalloproteinases (MMPs) leads to accumulation of collagen causing fibrosis [45, 48–51]. Three separate studies conducted by Tsai et al. revealed that interleukin-6, keratinocyte growth factor-1, and insulin-like growth factor-1 expressions were significantly upregulated in persons with OSF due to betel quid chewing and arecoline may be responsible for that [52–54]. A study conducted in India over a 10-year follow-up period has shown that the incidence of OSF was decreased by stopping areca nut use [55].

## 5. Link of Areca Nut to Other Diseases

Chewing areca nut can also cause various other diseases apart from malignant or potentially malignant disorders [41, 56]. Many studies have been conducted to find the relationship between various diseases and areca nut use. Arecoline is the most abundant alkaloid in areca nut [31]. It affects the autonomic nervous system and stimulates both sympathetic and parasympathetic nervous systems. It causes increase in pulse rate, dilation of the pupil, increase in peristalsis and tone in the intestine, and hyperthermic effect on skin temperature [16]. In addition, arecoline is cholinergic and decreases diastolic blood pressure [56]. Arecoline can cause placental damage and neonatal withdrawal syndrome if used by a pregnant woman [57]. Arecoline has the ability to interfere with adipose cell metabolism, thereby causing metabolic syndrome disorders [58]. It also causes bronchoconstriction and hence could affect asthma control and severity of attacks [59].

Studies have shown that arecoline inhibits growth and protein synthesis in periodontal fibroblasts suggesting that areca nut may exacerbate preexisting periodontal disease and impair periodontal reattachment [41, 60, 61]. Areca nut-induced lichenoid lesions have been reported at sites of quid application mainly on buccal mucosa or tongue [62]. This is considered as a type IV contact hypersensitivity reaction that resembles oral lichen planus clinically. Betel chewing can also cause brownish-red discoloration of the oral mucosa accompanied by encrustation of the affected mucosa with quid particles, with a tendency for desquamation and peeling which is known as chewer's mucosa [41].

## 6. Barriers to Cessation

A recent study done by Sotto et al., titled “Barriers to Quitting Areca Nut Consumption and Joining a Cessation Program as Perceived by Chewer and Nonchewer Populations in Guam” using 17 participants, categorizes the barriers to cessation into 3 main categories: sociocultural, behavioral, and accessibility [28].

Sociocultural influence refers to “the influence of members of the community, beliefs and attitudes that manifest through socialization and cultural relativity” [63]. It makes a chewer think that areca nut use is harmless. Betel chewing is a tradition in some cultures [63]. For example, Aboriginal Taiwanese population consider betel quid as a gift in a wedding ceremony. It is also used to build social interactions with guests during functions [64]. According to Paulino et al., Chamorro, Palauan, and Yapese betel quid chewers are proud of their habit as it improves social relationships. They also try to pass the habit to their younger generation [65]. A pilot study done on gutka users and non-users in Chitrakoot, India, concluded that the father of a family can influence children for gutka chewing involuntarily. There is a higher chance for children to become smokers if their parents and siblings smoke [27].

From ancient times, people in many Southeast and South Asian countries are used to chewing betel quid, and thus it has become a part of their culture [12]. There is historical evidence to suggest that in some societies areca nut is considered as a luxury food that only elders and individuals with authority in the society are allowed to consume [63]. Even though areca nut chewing is no longer limited to a certain social class in some societies, the habit of betel chewing is associated with a sense of belonging and acceptance. Sotto et al. mentioned that many participants reported that chewers prefer to chew when they are with close relatives and friends [28]. Some chewers reported that they do not encourage other chewers to go to cessation programs due to the fear of being labeled as a hypocrite. Little et al. reported that some chewers might fear the social consequences associated with quitting [66]. Those who are not willing to quit betel chewing do not like to see other people quitting the habit. They may manipulate ex-chewers to continue the habit and this will lead to relapses [28].

Sotto et al. revealed that the major challenges for chewers in Guam with attending cessation programs are time and transportation. Furthermore, participants explained that finding time to participate in a cessation program is difficult for the chewers who go to work on a daily basis [28]. Another study done in Guam discovered that some participants experienced language barriers during cessation programs [2].

Public misconceptions and lack of awareness and understanding of harmful effects of areca nut and importance of cessation programs have been identified as obstacles to areca nut cessation [28]. This finding has been supported by other studies done in Guam [65]. Some misconceptions are created by seeing other chewers. Sotto et al. reported that knowing about older people who have chewed areca nut during their entire life time without developing oral cancer, led some participants to think that areca nut is more beneficial than harmful. Some people believed that taking areca nut alone is not harmful, but rather adding tobacco and lime is the reason for development of cancer [28]. A study done among Burmese refugees in Australia revealed that they think betel chewing is not a harmful habit like smoking [67]. A study done using Cambodian refugees in USA also reported similar findings [68]. A pilot study done by Anwar et al. on attitudes and practices of gutka users and non-users in Chitrakoot, India, discovered that when compared to non-gutka users, a larger percentage of gutka users thought that gutka use is beneficial. They concluded that the lack of awareness regarding harmful effects of carcinogenic products is a serious problem due to the rising number of oral cancer cases in India [27]. A similar lack of awareness has been observed by Shetty and Johnson among the South Asian population in London [69].

Behavioral influence refers to “the sensations experienced while chewing areca nut and the actions taken in response to these sensations” [28]. Yang and Lin conducted research among taxi drivers in Taiwan to find out their experiences regarding areca nut chewing and quitting the habit. One major point revealed is that due to exhaustive nature of their work and prolonged working hours, they tend to use betel quid and other addictive substances to improve their physical strength, relieve their boredom, and maintain social relationships with other drivers [70]. The reason for the sensations experienced while chewing is the presence of chemicals in areca nut such as arecoline which causes neurological effects for the chewers such as providing a sense of euphoria, being alert and attentive, and feeling relaxed while chewing. Habitual influences such as chewing areca nut routinely at a specific time of the day also cause difficulty in stopping the habit [28].

Addiction or the compulsive desire to chew is another strong barrier to cessation [28]. Winstock et al. revealed that 10 out of 11 current and former heavy areca nut users reported cessation withdrawal effects such as mood swings, anxiety, irritability, reduced concentration, reduced energy, sleep disturbances, and increased appetite. They had a mean severity of dependence score of 7.3 consistent with the existence of a dependence syndrome among areca nut chewers [16]. The International Statistical Classification of Diseases and Related Health Problems 11^th^ Revision defines dependence syndrome as “disorder of regulation of use of a substance arising from repeated or continuous use of the substance. The characteristic feature is a strong internal drive to use the substance, which is manifested by impaired ability to control use, increasing priority given to use over other activities and persistence of use despite harm or negative consequences.” [71]. Chewers who use betel quid and areca nut products with tobacco have a greater dependence than chewers who use them without tobacco.

Accessibility refers to “ease of obtaining areca nut, lack of availability of information regarding harmful effects of areca nut consumption and difficulty to attend cessation programs” [28]. Availability of areca nut products can be reduced by making new policies. Although tobacco usage is restricted using various legislations and policies, including WHO Framework Convention on Tobacco Control (FCTC), there is no such policy to limit areca nut or betel quid usage [72]. Gutka, a commercial product which contains tobacco and areca nut, is banned in most of the states in India since 2012 under food safety regulations as it is illegal to add tobacco and nicotine to food [73]. Few studies [74–77] have assessed the effectiveness of banning and how it affects customers and sellers. Nair et al. revealed that the ban increased the awareness of smokeless tobacco (SLT) associated health effects on the public [76]. Mishra et al. reported that SLT users appreciated the ban as it persuaded them to stop or control the habit [75]. A study done in 2014 discovered that the gutka ban has actually reduced the use of gutka but they worried that they may have found alternative products to use [78].

These barriers should be eliminated with proper planning in order to reduce the number of areca nut chewers in the society. Public misconceptions, lack of awareness, and sociocultural influence can only be addressed by proper awareness programs targeting individuals as well as the society. Mass media campaigns, awareness programs, school based educational programs, and poster campaigns can be used to increase the public awareness regarding harmful effects of areca nut chewing [14, 72, 79, 80]. Religious leaders and celebrities can be accompanied to promote the message. Policies should be implemented to decrease the availability of areca nut and commercial products [73]. The number of cessation programs should be increased, and they should be held at a convenient time to the participants. Transport facilities can be provided or an allowance can be paid. Awareness and cessation programs should be available in all local languages. [2].

## 7. Cessation Programs

A review done by Das et al. has identified eight studies regarding areca nut and betel quid cessation [2, 80–86]. Out of them, only two studies are directly related to areca nut cessation [2, 83]. Other studies are designed mainly focusing on tobacco cessation [72]. This emphasizes the serious lack of studies on areca nut cessation.

Due to lack of studies regarding areca nut cessation, a cessation program was modeled for Island of Guam as a feasibility study using the protocol of an intensive behavioral treatment program for smokers presented in *The Tobacco Dependence Treatment Handbook: A Guide to Best Practices* [87]. It is a group based cognitive-behavioral cessation program. A total of 50 participants were recruited for the program, and they were divided into groups of 5–10 people. Participants in one group took part in five one-hour sessions facilitated by one of the study investigators over a period of 22 days. First two sessions were aimed at preparing the participants for quitting by self-monitoring the chewing habit, reducing chewing rate, and identifying situations that trigger the temptation to chew. At the beginning of session 3, participants discussed their quitting experiences including the challenges, coping with withdrawal symptoms, and avoiding relapses [2].

Lack of awareness, scheduling problems, lack of contribution of female members in the family, inexperience of the facilitator with betel quid, lack of social support, financial difficulties for participants in rewarding themselves for remaining abstinent, increased food consumption after quitting, and language barriers were some of the problems identified during the program [2].

Betel Nut Intervention Trial (BENIT), the first known randomized trial aimed at areca nut chewing cessation, was implemented recently in Guam and Saipan [88]. Previous studies conducted in those 2 islands revealed that prevalence of OPMDs was higher in chewers who use betel quid/areca nut with tobacco and slaked lime (class 2) than in chewers who use areca nut alone or with betel quid without tobacco (class 1) [88]. Therefore, class 2 chewers were the target population of the study.

Initially, 324 participants were chosen for the research, and they were divided into intervention and control groups. The participants in the intervention group received an educational brochure and a cessation program whereas the participants in the control group received only the educational brochure. The booklet entitled *Quitting Betel Nut* was specifically designed for this study to encourage participants in areca nut cessation. It contained general information on areca nut and risks associated with areca nut chewing emphasizing the importance of cessation and cessation strategies.

Intervention was carried out as a cognitive-behavioral therapy. The cognitive component addressed areca nut users' perspective on areca nut chewing by educating them. The objective of the behavioral component is to eliminate behaviors which promote chewing by identifying and managing triggers, making lifestyle changes that support quitting and preparing participants to face unsupportive social situations. The intervention consisted of five-session support groups held within 22 days. Intervention groups included up to 10 members, and individual based option was available for the participants who wished to participate alone. The meetings were 1 hour long, and surveys were given at the beginning of the first and the last meetings and six months after the last meeting.

Saliva samples were collected at the beginning, after 22 days and after 6 months to verify cessation self-reports from the participants from both groups [89]. Results of this study are still pending.

Another study related to betel quid cessation was conducted by Lee et al. in Taiwan using transtheoretical model framework to discover behavioral changes of areca nut chewers [83]. 30 oral cancer patients who had the habit of betel quid chewing were selected for the study. Data were collected through interviewer administered questionnaires. Investigators found that the participants typically undergo four stages of behavior status: precontemplation, contemplation, action, and maintenance. The participants in precontemplation stage did not believe that betel chewing has adverse effects, and thus they did not have the motivation to stop the habit. However, the participants in contemplation stage believed that betel chewing has adverse effects on their appearance and oral health even though it would not impair their social interactions. When the participants decided to give up the habit, most of them reported quitting betel chewing completely at once and practicing self-restraint [83].

Most of the participants of this study were motivated to stop betel chewing after being informed that they suffer from cancer or precancerous lesions. Some participants quit the habit because of inability to obtain betel quid and some due to the loss of oral functions because of the disease. Participants who quit the habit due to oral cancer or poor oral functioning had a strong willpower and succeeded in quitting the habit. On the other hand, some participants experienced relapses during maintenance stage due to stress, social pressure, and withdrawal symptoms [83].

Hussain et al. have done a randomized controlled trial on 2140 adolescents from schools in Karachi, Pakistan, focusing on school based behavior changing intervention (BCI) to stop the habitual use of SLT and BQ in adolescents [80]. The results from the study were promising, and the attitudes regarding SLT and BQ use and cessation were changed in both users and non-users significantly [80].

The study concluded that school based BCI is a successful way to increase the awareness of school-going adolescents regarding the harmful effects of SLT and BQ use, and such programs motivate SLT and BQ users to quit the habit. The study also proposed to include such awareness programs in the school curricula [80].

Another study done by Siddiqi et al. in 2016 involving 32 SLT users in Pakistan and United Kingdom focused on BCI and showed promising results [86]. All participants reported that they felt highly motivated to quit SLT after the prequit session because of the facts they learned during the program. Most participants found that information regarding potential triggers for using SLT and ways to eliminate them was useful in quitting the habit. Negative portrayal of SLT users and photographs of the patients with diseases caused by SLT use were also found to be compelling and motivating. Counsellors in UK were hesitant to advise the patients to quit the habit completely, due to the fear of losing clients [86].

At the moment, there is not any pharmacologically based cessation therapy available to aid in areca nut cessation as in tobacco cessation. However, a double blinded randomized clinical trial was done in 2019 by Hung et al. to find out the effectiveness of antidepressants for cessation therapy in betel quid use disorder (BUD) [90]. 111 male patients with BUD were randomly divided into 3 drug treatment groups during the study. Patients in group A were treated with a placebo while patients in B and C groups were given escitalopram and moclobemide, respectively. Progress of the patients was evaluated at 2-, 4-, 6-, and 8-week intervals. BQ intake among participants treated with escitalopram and moclobemide was found to decrease with increase in treatment time. The proportion of betel quid cessation in patients who received placebo, escitalopram, and moclobemide treatments was 5.4, 34.2, and 33.3%, respectively. This study concluded that BUD patients respond to pharmacotherapy, and prescribing a fixed dose of escitalopram and moclobemide to BUD patients over 8 weeks is beneficial. The study also recommended that more studies should be carried out in the future, building upon their findings [90].

## 8. Conclusion

Studies regarding areca nut cessation should be given proper attention as areca nut contains many chemicals which are harmful to the human body. As a result of various reasons including cultural beliefs, social misconceptions, lack of policies, and lack of research, many people are unaware of the possible harmful effects of areca nut. In addition, only few studies with regard to areca nut cessation can be found in the literature. The findings of the limited studies that have been conducted reveal the fact that behavior controlling interventions are more effective in areca nut cessation. Some areca nut chewers have reported relapses after cessation due to withdrawal symptoms, peer pressure, and behavioral factors. Lack of awareness, sociocultural influence, and issues of accessibility can be identified as the main reasons for failures of cessation programs. Making cessation programs easily accessible, increasing awareness, involving family in sessions, making learning material available in local languages, including pictures of patients with oral cancer, having an ex-chewer as an advisor, giving financial support, and increasing social support are some of the things recommended by previous studies on cessation to improve future cessation programs.
